# Efficacy and Safety of Dual Paclitaxel and Sirolimus Nanoparticle-Coated Balloon

**DOI:** 10.1016/j.jacbts.2024.02.002

**Published:** 2024-05-01

**Authors:** Kenji Kawai, Mohammed Tanjimur Rahman, Ryan Nowicki, Frank D. Kolodgie, Atsushi Sakamoto, Rika Kawakami, Takao Konishi, Renu Virmani, Vinod Labhasetwar, Aloke V. Finn

**Affiliations:** a CVPath Institute, Gaithersburg, Maryland, USA; b Department of Biomedical Engineering, Lerner Research Institute, Cleveland Clinic, Cleveland, Ohio, USA; c Advanced NanoTherapies, Inc, Los Gatos, California, USA; d University of Maryland, School of Medicine, Baltimore, Maryland, USA

**Keywords:** downstream embolization, drug-coated balloon, myocardium injury, preclinical research, vascular response

## Abstract

We evaluated a novel dual active pharmaceutical ingredient (API) drug-coated balloon (DCB), which consists of a coating of nanoparticles encapsulating low-dose paclitaxel (PTX) in combination with sirolimus in a synergistic ratio. Compared to the PTX DCB, the dual API DCB demonstrated similar inhibition of cell proliferation in vitro but at a significantly lower total drug dose (over 13 times lower than sirolimus nanoparticles). Animal experiments demonstrated that the dual API DCB is more effective in inhibiting intimal cell proliferation with insignificant downstream embolic effects and myocardial damage compared to the PTX DCB. These findings indicate that dual API DCBs have a high potential to demonstrate improved clinical outcomes and a greater safety profile than the PTX DCBs.

## Highlights

• Dual API NPs, with a low dose of PTX in combination with SRL, demonstrated a synergistic effect in inhibiting VSMC proliferation via cell cycle arrest.
• Dual API DCB–treated arteries in rabbit iliac model demonstrated a significantly lower percent of BrdU-positive nuclei in intima compared to PTX DCB–treated arteries at 5 days.
• Dual API DCB–treated arteries displayed notably less VSMC loss than PTX DCB-treated arteries at 28-day follow-up.
• In porcine coronary model, PTX DCB caused significantly greater myocardial injury and downstream embolism, whereas with dual API DCB there was no myocardial injury and infrequent incidence of downstream embolism.
• Innovative dual API DCB demonstrated superior efficacy in suppressing intimal proliferation with minimal vascular injury and less downstream myocardial tissue injury.

## Visual Abstract

**Figure:**
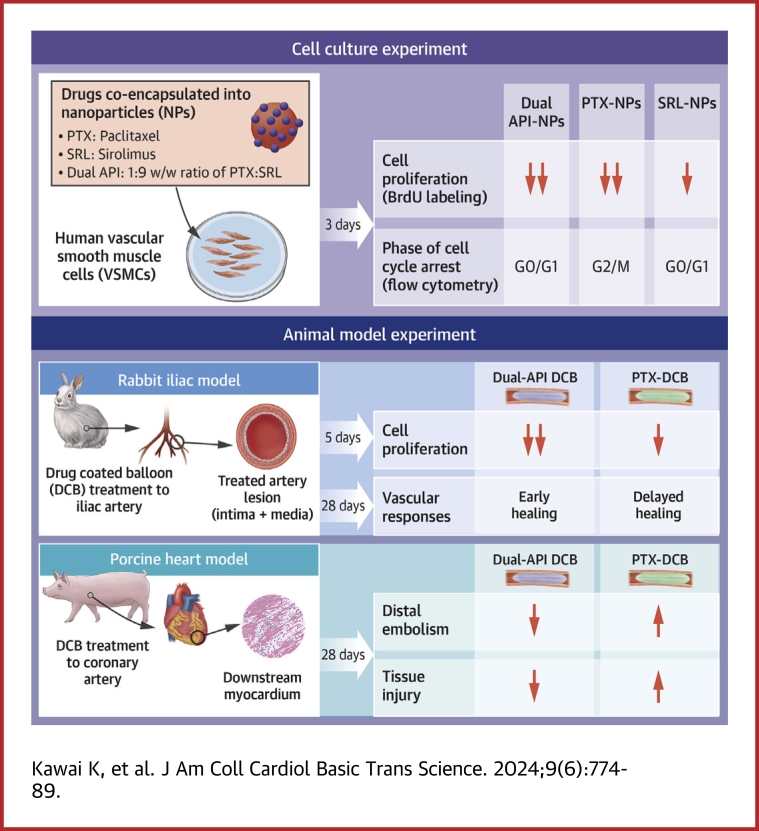

Drug-coated balloons (DCBs) are an emerging option for the treatment of obstructive arterial diseases.^1^^,^^2^ However, inefficiency of drug delivery to the target artery results in an increased risk of embolization and inflammation in downstream tissues.^3^, ^4^, ^5^ This is caused by large-sized drug crystals and the coating materials used, which can block blood vessel capillaries.^5^^,^^6^ Moreover, most DCBs use paclitaxel (PTX), a highly toxic drug that can cause tissue necrosis.^7^^,^^8^ Sirolimus (SRL) is considered a safer drug than PTX because of its cytostatic mechanism of inhibiting cell proliferation, but its efficacy in randomized clinical trials is yet to be established.^9^

PTX is a cytotoxic drug known to strongly suppress neointimal formation. Although clinical studies have demonstrated safety at long-term follow-up of PTX DCBs,^10^^,^^11^ medial vascular smooth muscle cell (VSMC) loss and at higher doses medial wall necrosis have been reported in animal models and in some human studies.^4^^,^^12^^,^^13^ On the other hand, SRL is a cytostatic drug that effectively prevents intimal formation after arterial injury with a much wider therapeutic index but has limited arterial wall residence because of its relatively lower lipophilic nature and affinity to tissue proteins than PTX.^14^^,^^15^ Although each drug has its advantages and limitations when delivered to the arterial wall, we explored the concept of combining both the drugs, PTX and SRL, into 1 device in a synergistic combination in anticipation that it would result in superior efficacy and improved safety if the dose of PTX is significantly low compared to what is loaded onto conventional PTX DCBs.

This study evaluated SirPlux Duo (Advanced NanoTherapies, Inc), a dual active pharmaceutical ingredient (API) DCB in which PTX and SRL are coencapsulated (1:9 w/w ratio) into sustained release, biodegradable nanoparticles (NPs). The combination was optimized to enhance the antiproliferative effect while keeping the cytostatic mechanism of inhibition of cell proliferation. The NPs are surface functionalized with poly-L-lysine to enhance cellular uptake and retention.^16^ This innovative dual API NP coating technology offers potential benefits, including a reduced risk of cytotoxicity and distal embolization due to nanometer size range of the coated NPs as well as their sustained release property. This study investigated the performance of the dual API DCB for its vascular response in a rabbit iliac artery model and embolization into the downstream myocardium in a porcine coronary model compared to commercially available PTX DCBs.

## Methods

### In vitro cell culture studies

Three sets of experiments were performed in human VSMCs (Cat# 354K-05a, Cell Applications Inc). Cells were cultured in a smooth muscle cell growth medium (Cat# 311-500, Cell Applications Inc) at 37 °C in a humidified 5% CO_2_ atmosphere. After the initial experiment determined that the 1:9 w/w ratio of PTX:SRL is highly synergistic (details are described in the Supplemental Methods), the following 2 experiments were conducted.

#### Analyzing dead/live cells and bromodeoxyuridine-positive proliferating cells

Thirty thousand cells per well were seeded in each well of 8-well LAB-TEK chamber slides (Cat# 154534, Thermo Fisher Scientific). A day after cell seeding, cells were treated with NPs encapsulating SRL, PTX, or a combination of PTX and SRL (1:9 w/w ratio, dual API NPs). Three days after the incubation period, a percentage of apoptotic/dead cells with respect to the total number of cells was calculated and compared among the 3 groups (details are described in the Supplemental Methods).

#### Cell cycle analysis with bromodeoxyuridine-fluorescein isothiocyanate (FITC) flow cytometry assay

Cells cultured as described previously were seeded into 6-well plates (Cat# 3516, Corning Inc) at a density of 150,000 cells/well. The following day cells were treated at the half-maximal inhibitory concentrations (IC_50_) as described in experiment 1 and incubated for 3 days. Cells were incubated with 10 μmol/L bromodeoxyuridine (BrdU) for 12 hours before cell harvesting. After the incubation period, media from the wells were collected in separate tubes for apoptotic floating cells followed by dissociation of the adherent cells using Accutase (Cat# A6964, MilliporeSigma). The samples were prepared for flow cytometry cell cycle analysis using the BD Pharmingen FITC BrdU Flow Kit (Cat# 557891). The samples were run through the BD LSRFortessa flow cytometer (BD Biosciences), and the data were analyzed using FlowJo software (BD Biosciences).

### In vivo study design

The study protocols were approved by the Institutional Animal Care and Use Committee of the MedStar Health Research Institute and American Preclinical Servies. Three different animal model experiments were performed to compare the vascular response to treatments with the dual API DCB and its effects on downstream tissues in comparison with the PTX DCB (Supplemental Figure 1). These studies were performed in healthy animal models. The dual API DCB used was SirPlux Duo for both the experiments, whereas the PTX DCB used was the Prevail (Medtronic) for experiment 2 (ie, the rabbit iliac artery model) and the Agent (Boston Scientific) for experiment 3 (ie, the porcine coronary artery model). Detailed descriptions of each of these devices are provided in Supplemental Table 1.

### Experiment 1: swine femoral artery drug uptake and retention

Three adult domestic pigs (35-45 kg) were examined. One dual API DCB was deployed at 2 to 4 treatment sites per animal (the right and left internal and external femoral arteries). The target balloon-to-artery ratio was 1.1:1, and the balloon inflation time was 180 seconds. Femoral arterial samples were obtained after euthanasia at 1 day and 28 days after treatment. Each sample was homogenized, extracted, and analyzed for drug levels using liquid chromatography mass spectroscopy at contract analytical laboratories.

### Experiment 2: rabbit iliac model with 5- and 28-day follow-up

#### Procedures

Nine New Zealand White male rabbits were used (2.7-4.0 kg, Charles River Laboratories). Animals were pretreated with aspirin (40 mg) 1 day before the initial procedure. Under general anesthesia, the right and left iliac arteries were injured by endothelial denudation using a 3.0 × 8 mm balloon via transcatheter approach as per a previous study.^17^ Subsequently, treatment was performed with a 3.0 × 20 mm dual API DCB in the unilateral iliac and a PTX DCB (Prevail, Medtronic) in the contralateral iliac. The balloon inflation time was set at 180 seconds for each device, and the target balloon-to-artery ratio was set at 30% oversize (ranging from 1.25:1 to 1.35:1 based on the balloon compliance chart). Five and 4 animals were assigned to 5- and 28-day time points, respectively. Animals in all the study groups survived the planned study duration. All the arterial tissues were subsequently processed for light microscopy and immunohistochemical evaluation. The animals at the 5-day time point were injected twice with BrdU (50 mg/kg) 17 to 20 hours and 2 to 4 hours before being sacrificed. BrdU was used as a marker for proliferating (DNA replication) in living tissue.

#### Tissue processing and histology preparation

The treated iliofemoral arteries were carefully dissected free from surrounding tissue and prepared histology sections with hematoxylin and eosin (H&E), Movat pentachrome stains, and BrdU (Cat# MO744, DAKO/Agilent) (details in Supplemental Methods).

#### Morphometry, histology, and cell proliferation analysis

For morphometric and histologic evaluation, 3 arterial sections showing the most severe changes consistent with drug treatment effects were evaluated. All histology parameters were semiquantified using a 4-point scoring system according to a previous publication.^12^ The number of total nuclei and the BrdU-positive nuclei were counted in the intima and media separately using HALO imaging software (Indica Labs, Corrales). The index of BrdU-positive cells was expressed as a ratio of (chromophore-positive nuclei/total nuclei) × 100 (percentage of BrdU) and was calculated for intimal and medial regions of the artery (details in the Supplemental Methods).

### Experiment 3: swine myocardium at 28-day follow-up

#### Coronary artery catheterization and intervention

Four healthy Yorkshire cross female domestic pigs (35-55 kg, Thomas D. Morris, Inc) were used. Animals were pretreated with aspirin (324 mg) and clopidogrel sulfate (75 mg) for 3 days before the initial procedure and received dual antiplatelet therapy with aspirin (81 mg/d) and clopidogrel (75 mg/d) after the treatment procedure and continued for the duration of the study. At the initial procedure after the endothelial denudation using a plain uncoated balloon at the selected sites, the dual API DCB or the PTX DCB (3.0 × 20 mm) was deployed at the target site in the left anterior descending or left circumflex artery, and plain old balloon angioplasty (POBA) (3.0 × 20 mm) was deployed at the target site in the right coronary artery (details in the Supplemental Methods). Each balloon was inflated for 60 seconds with a target balloon-to-vessel ratio of at least 10% based on a compliance chart that achieved a range of 0.99:1 to 1.3:1.

#### Tissue processing and assessment of downstream myocardium

Animals underwent a final follow-up angiography on day 28 and were sacrificed (details in the Supplemental Methods). After the termination, a coronary artery sample was harvested from the treated segment, prepared, and assessed for vessel morphometry and histologic scoring with the same method as described for the rabbit model. A myocardium sample was sliced from the heart in the short axis at a thickness of 1.0 to 1.5 cm parallel to the atrial groove. For each region of downstream myocardium, full-thickness myocardial sections downstream to the balloon-treated arteries at the mid and distal levels were circumferentially sampled in the distribution of the major coronary arteries (anterior, lateral, posterior, and septal wall). In addition, the samples were also taken at 2 nearby locations in the areas near the treated coronary arteries (myocardium 1 [MYO 1] and myocardium 2 [MYO 2]). The sections of downstream myocardium corresponding to the treated coronary arteries are shown in Supplemental Table 2. The samples were then dehydrated in a graded ethanol solution, embedded in paraffin, sectioned at 4 to 6 μm with a rotary microtome, and then stained with H&E. Histology sections for each downstream myocardium were assessed with a light microscope for tissue injury (ie, necrosis, scarring, and granulation) and the presence of potential foreign materials. The number of histology sections with these findings between each device was counted, and the percentage of the total number of regions was calculated.

### Statistical analysis

For histomorphometry and histology scores, the mean values per artery were calculated for the 3 selected tissue sections. Categoric variables are presented as counts and percentages and continuous variables as medians with 25th to 75th percentiles (Q1-Q3) unless otherwise specified. Categoric variables were compared using the chi-square test, whereas continuous variables were compared using the Student’s *t*-test or the Wilcoxon rank sum test for 2 groups based on data normality. An analysis of variance or Kruskal-Wallis test, respectively, was used to compare 3 groups based on data normality. The normality of the distribution was determined using the Shapiro-Wilk test. Statistical analyses were performed using JMP software (Version 15.0, JMP Statistical Discovery LLC). *P <* 0.05 was considered statistically significant.

## Results

### Effect of different treatments on cell proliferation

**Figure 1:**
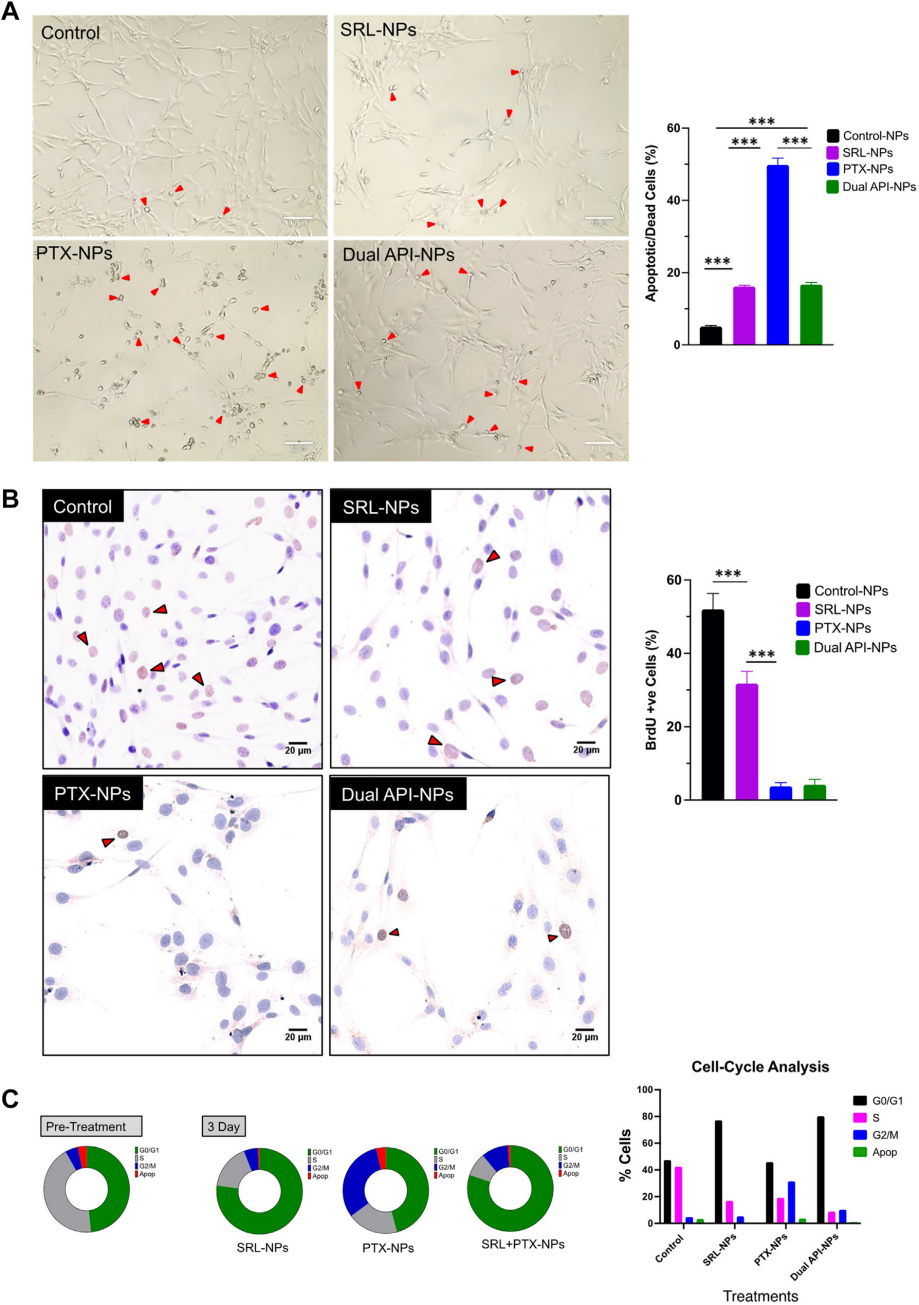
In Vitro Cell Culture Studies Human vascular smooth muscle cells were treated at IC_50_ (ie, the dose required to achieve 50% inhibition of cell proliferation with respect to untreated cells) drug doses in nanoparticles (NPs) for 3 days. Different treatments were control NPs, sirolimus (SRL) NPs (3,100 ng/mL), paclitaxel (PTX) NPs (45 ng/mL), and dual active pharmaceutical ingredient (API) NPs (225 ng/mL). (A) Microscopic observation and quantitative analysis of apoptotic/dead cells following treatment with control NPs, SRL NPs, PTX NPs, and dual API NPs. Red arrowheads indicate the apoptotic/dead cells. Bar = 10 μm. The data show significantly higher apoptotic/dead cells in PTX NPs than in other treatment groups. (B) Quantification of proliferating cells. The BrdU-positive cells following different drug treatments were imaged and quantified. Red arrowheads in the images show proliferating cells. Bar = 20 μm. The quantitative analysis shows a significantly lower number of bromodeoxyuridine (BrdU)-positive cells in dual API NPs compared to control or SRL NPs. (C) Flow cytometric analysis. Cells following different treatments were analyzed for different phases of cell cycle. The results show that dual API NPs cause the inhibition of cell proliferation by cell cycle arrest (G0/G1). Different phases of the cell cycle. G0/G1 phase = cells exit G1 and enter a resting state/cell cycle arrest phase called G0 (SRL mechanism); G2/M phase = gap 2 (G2); M phase= mitosis (PTX mechanism); S phase = DNA synthesis for cell replication or proliferative phase. Values are mean ± SEM. Differences in the groups were calculated using the Student’s *t*-test. ∗∗∗*P* = 0.001.

The experiments in human VSMCs were performed at IC_50_ drug doses (ie, the dose that results in the inhibition of cell proliferation by 50% following treatment over 3 days). Accordingly, the drug dose for SRL was 3,100 ng/mL; for dual API, it was 225 ng/mL (22.5 ng PTX + 202.5 ng SRL); and for PTX, it was 45 ng/mL. The percentage of apoptotic/dead cells with respect to the total number of cells computed for different treatments was similar for SRL NPs and dual API NPs; however, this number was significantly higher for PTX NPs (Figure 1A). The BrdU results showed a reduced number of proliferating cells that were treated with SRL NPs compared with control NPs; however, there were significantly fewer proliferating cells in dual API NP– and PTX NP–treated cells than those treated with SRL NPs. The low percentage of proliferating cells in PTX NPs is because a larger number of apoptotic/dead cells were washed off before they were fixed for imaging to quantitate the number of BrdU-positive cells (Figure 1B). The flow cytometric analysis showed that a majority of the cells treated with SRL NPs and dual API NPs were in the G0/G1 cell cycle arrest phase, whereas the cells treated with PTX NPs were in the G2/M phase. The dual API NPs also showed a lower number of cells in the proliferative S phase than in the other treatment groups (Figure 1C, Supplemental Table 3).

### Pharmacokinetic evaluation in porcine femoral arteries

All the dual API DCBs were successfully deployed without dissection or thrombosis. The median (Q1-Q3) measured arterial tissue total drug concentrations of combined SRL and PTX at days 1 and 28 were 4.86 ng/mg (Q1-Q3: 4.22-5.23 ng/mg) and 0.89 ng/mg (Q1-Q3: 0.73-1.19 ng/mg), respectively (Table 1). The ratio of SRL to PTX at 28 days was 9.24:0.86, which was close to the ratio of the 2 drugs (9:1) used for loading onto dual API DCBs.

**Table 1:** Sirolimus and Paclitaxel Concentrations in Porcine Iliac Arterial Tissue at 1 Day and 28 Days

Vessel	SRL (ng/mg)	PTX (ng/mg)	Total (ng/mg)
1 Day			
REF	4.16	1.22	5.38
LEF	3.51	0.67	4.18
LIF	4.58	0.78	5.36
RIF	2.86	1.49	4.35
Median (Q1-Q3)	3.84 (3.02-4.39)	1.00 (0.70-1.37)	4.86 (4.22-5.23)

28 Days			
REF	0.68	0.04	0.72
RIF	0.91	0.12	1.03
REF	0.67	0.07	0.74
RIF	1.17	0.07	1.24
Median (Q1-Q3)	0.80 (0.67-1.11)	0.07 (0.05-0.11)	0.89 (0.73-1.19)

LEF = left external femoral artery; LIF = left internal femoral artery; PTX = paclitaxel; REF = right external femoral artery; RIF = right internal femoral artery; SRL = sirolimus.

### Vascular response at 5 and 28 days in the rabbit iliac model

#### Vascular response at 5 days

All 5 animals survived until the scheduled 5-day study time point. A comparison was performed between 5 arteries treated with the dual API DCB and 5 arteries treated with the PTX DCB. Table 2 shows the overall results of the morphologic and histologic analysis. The dual API DCB–treated group showed less neointimal area than the PTX DCB–treated group (median [Q1-Q3]: 0.0 [0.0-0.001] mm^2^ vs 0.03 [0.0-0.04] mm^2^; *P =* 0.061). A similar trend was observed for percent stenosis (median [Q1-Q3]: 0.0% [0.0%-0.2%] vs 1.7% [0.2%-2.7%]; *P =* 0.089) and neointimal thickness (median [Q1-Q3]: 0.0 [0.0-3.0E-10] vs. 0.01 [0.0-0.02] mm; *P =* 0.069). There were no significant differences in other morphologic parameters between the 2 devices (Table 2). For the histologic semiquantification at 5 days, the endothelial cell (EC) loss score was significantly greater in the dual API DCB group than in the PTX DCB group (median [Q1-Q3]: 4.0 [4.0-4.0] vs 2.3 [1.7-3.7]; *P =* 0.018), but other parameters were not significantly different between the 2 devices. Both groups showed more than mild changes of proteoglycan (PG) and collagen deposition in media (median [Q1-Q3]: 2.0 [1.8-2.3] vs. 2.0 [0.8-3.2]; *P =* 0.92) and more than moderate changes for the loss of smooth muscle cell in depth (median [Q1-Q3]: 3.3 [3.0-4.0] vs 4.0 [2.7-4.0]; *P =* 0.91). Both groups had no findings for medial and neointimal fibrin and calcification (Table 2). These morphologic and histologic findings showed that there was less of a trend for neointimal proliferation at 5 days in the dual API DCB–treated group than the PTX DCB–treated group, with similar histologic injury between the 2 groups with the exception of EC loss.

**Table 2:** Morphometric and Histologic Results at 5 and 28 Days in the Rabbit Femoral Artery Model

	5 Days			28 Days		
	Dual API (n = 5)	PTX CTL (n = 5)	*P* Value	Dual API (n = 4)	PTX CTL (n = 4)	*P* Value
Morphometry						
EEL area, mm^2^	2.5 (2.4-3.0)	2.3 (1.7-2.8)	0.23	1.9 (1.6-2.7)	2.5 (2.4-2.6)	0.24
IEL area, mm^2^	2.0 (1.9-2.4)	1.8 (1.3-2.5)	0.43	1.5 (1.2-2.1)	2.1 (2.0-2.2)	0.15
Lumen area, mm^2^	2.0 (1.9-2.5)	1.8 (1.3-2.5)	0.37	1.2 (0.8-1.8)	1.7 (1.5-1.8)	0.20
Medial area, mm^2^	0.4 (0.3-0.4)	0.4 (0.3-0.4)	0.51	0.4 (0.4-0.5)	0.4 (0.4-0.4)	0.36
Neointimal area, mm^2^	0.0 (0.0-0.001)	0.03 (0.0-0.04)	0.061	0.4 (0.4-0.4)	0.4 (0.3-0.4)	0.98
% Stenosis, %	0.0 (0.0-0.2)	1.7 (0.2-2.7)	0.089	23.9 (19.2-35.9)	19.3 (14.8-22.4)	0.20
THK mean, mm	0.0 (0.0-3.0E-10)	0.01 (0.0-0.02)	0.069	0.1 (0.1-0.2)	0.1 (0.1-0.1)	0.98
Histology						
EC loss	4.0 (4.0-4.0)	2.3 (1.7-3.7)	0.018	0.0 (0.0-0.0)	0.0 (0.0-0.3)	0.32
Fibrin/platelet thrombus (surface)	0.0 (0.0-0.2)	0.0 (0.0-0.8)	0.28	0.0 (0.0-0.0)	0.0 (0.0-0.0)	1.00
Medial/neointimal fibrin	0.0 (0.0-0.0)	0.0 (0.0-0.7)	0.32	0.0 (0.0-0.0)	0.0 (0.0-0.0)	1.00
Proteoglycan/collagen (media)	2.0 (1.8-2.3)	2.0 (0.8-3.2)	0.92	0.0 (0.0-0.5)	1.5 (0.3-2.2)	0.093
Inflammation (intima/media)	1.7 (1.5-2.0)	1.0 (0.8-1.8)	0.19	0.1 (0.0-0.6)	0.8 (0.2-1.0)	0.18
Calcification	0.0 (0.0-0.0)	0.0 (0.0-0.0)	1.00	0.0 (0.0-0.0)	0.0 (0.0-0.0)	1.00
Medial SMC loss (depth)	3.3 (3.0-4.0)	4.0 (2.7-4.0)	0.91	0.0 (0.0-0.0)	2.5 (0.4-3.8)	0.047
Medial SMC loss (circumference)	2.67 (2.5-3.5)	1.7 (1.2-3.0)	0.12	0.0 (0.0-0.0)	1.3 (0.2-2.0)	0.045

Values are median (25th-75th percentile).

CTL = control; EC = endothelial cell; EEL = external elastic lamina; IEL = internal elastic lamina; SMC = smooth muscle cell; THK = thickness of neointima.

#### Cell proliferation in intima and media at 5 days

**Figure 2:**
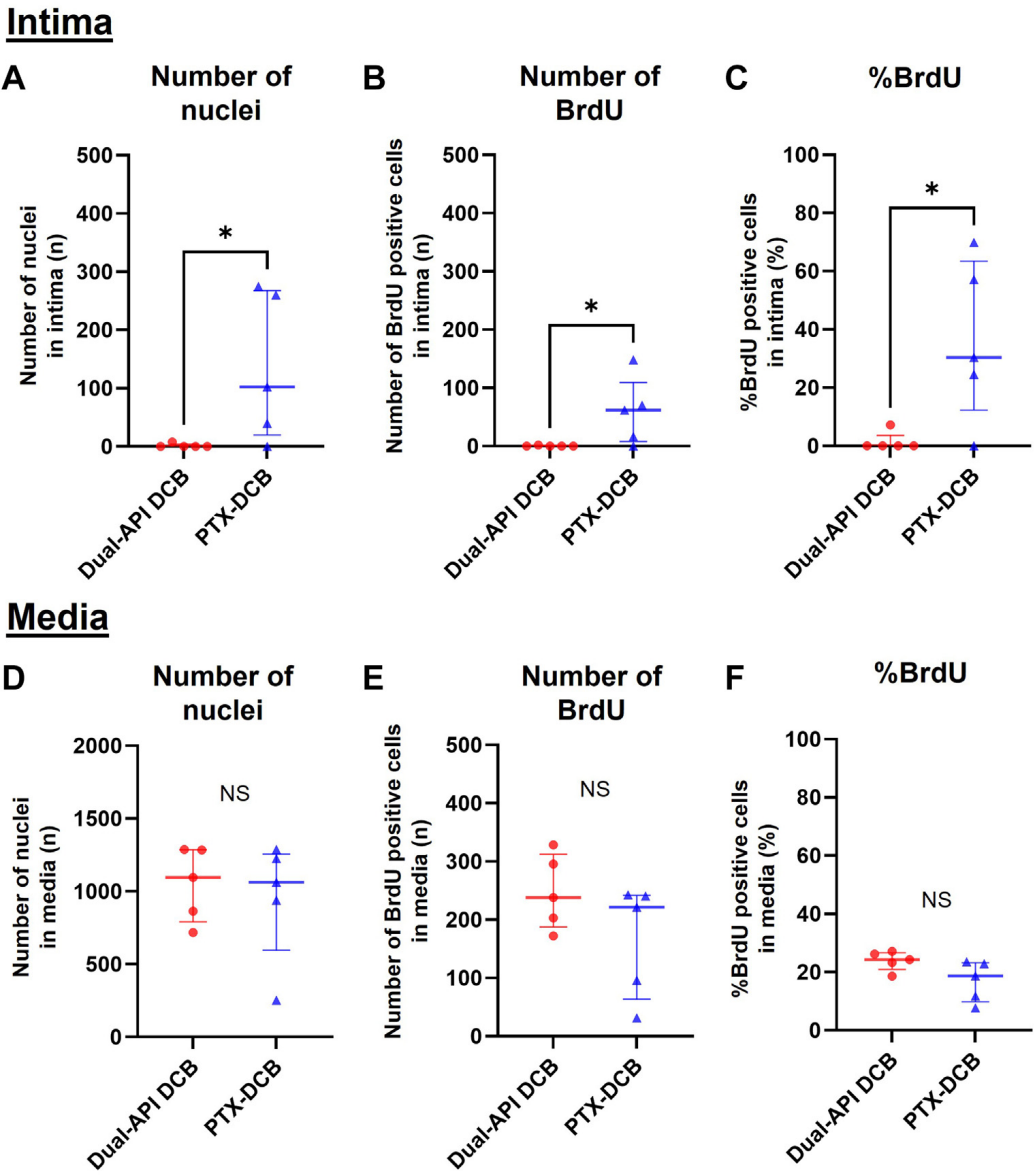
Assessment of Cell Proliferation in the Neointima and Media of Dual API DCB– or PTX DCB–Treated Rabbit Iliac Arteries by BrdU Staining at 5 Days The total number of nuclei (A) in the neointima and (D) in the media, the number of BrdU-positive nuclei (B) in the neointima and (E) in the media, and the percentage of BrdU-positive cells (C) in the neointima and (F) the media. Three arteries were used for assessment per treatment group. The number of nuclei and the number of BrdU-positive nuclei showed no significant difference between the 2 groups in both the intima and media. The percent of BrdU-positive cells in the intima was significantly lower in the artery treated with the dual API drug-coated balloon (DCB) compared with the PTX DCB, but there was no significant difference between them in the media. Statistical analyses were performed using the Wilcoxon rank sum test for A to C and the Student’s *t*-test for D to F. ∗*P <* 0.05. Abbreviations as in Figure 1.

**Figure 3:**
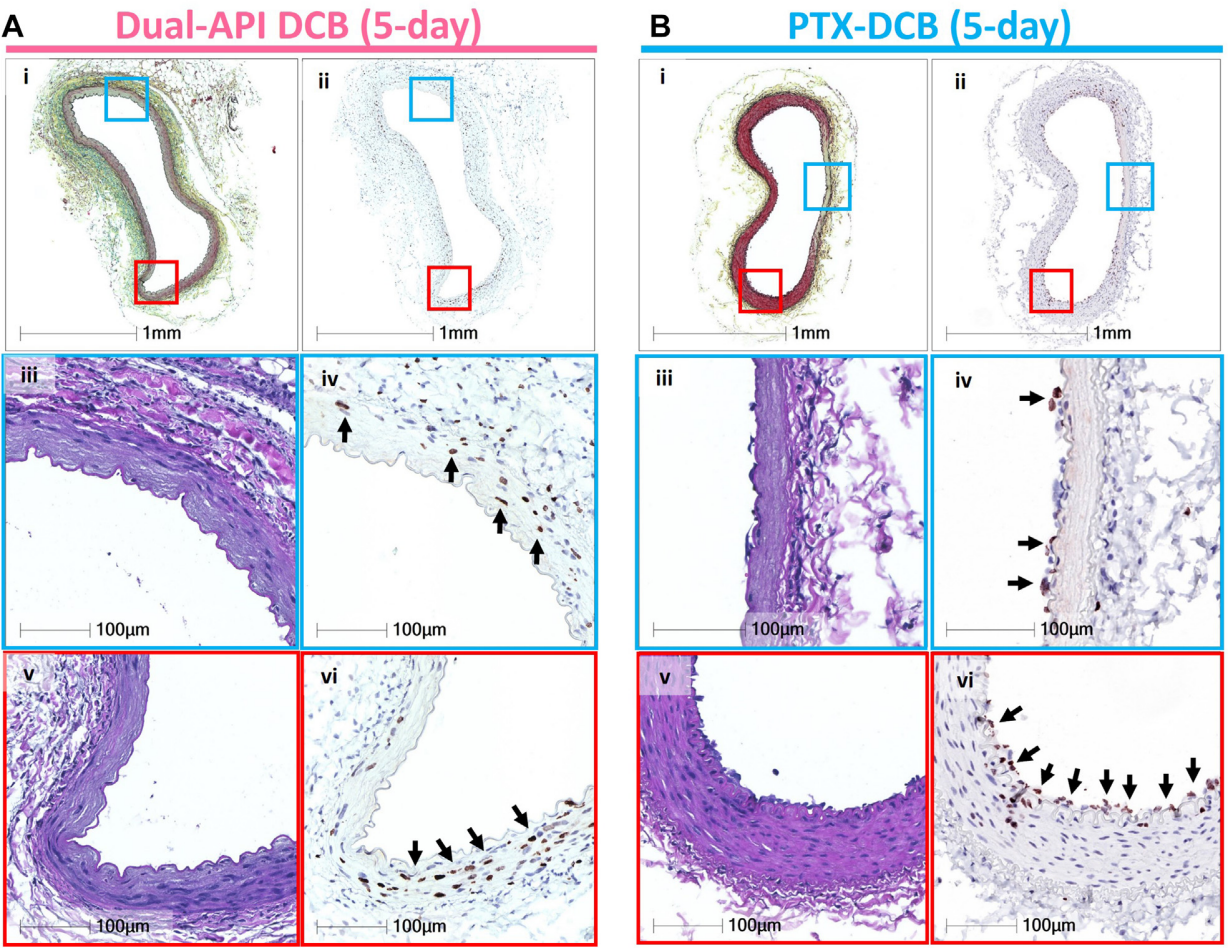
Representative Histology of Rabbit Iliac Arteries 5 Days After the Treatment With Dual API DCB or PTX DCB Histology sections from iliac arteries treated with (A) the dual API DCB or (B) the PTX DCB. (Upper) Low-power images of (A-i and B-i) Movat pentachrome-stained and (A-ii and B-ii) BrdU-stained images. (Middle) High-power images of (A-iii and B-iii) hematoxylin and eosin (H&E) and (A-iv and B-iv) BrdU staining from blue rectangular boxes seen in upper images. (Bottom) High-power images of (A-v and B-v) H&E and (A-vi and V-vi) BrdU staining from red rectangular boxes in upper images. There is (A-iii and A-v) smooth muscle cell (SMC) loss with (A-iv) BrdU-positive nuclei (arrows) in the media of the section treated with the dual API DCB, whereas the section treated with the PTX DCB showed (B-iii) severe loss of SMC without (B-iv) BrdU-positive nuclei in media. (A-iii to A-vi) There were no nuclei on the luminal surface in the section treated with the dual API DCB, whereas (B-iv and B-vi) BrdU-positive nuclei were observed in the neointimal area of the section treated with the PTX DCB (arrows). Abbreviations as in Figure 1.

The results of cell proliferation analysis by assessing BrdU-positive nuclei at 5 days after treatment are shown in Figure 2. In the neointima, the median number of nuclei was 0 for the dual API DCB, a significantly lower value compared to the PTX DCB (median [Q1-Q3]: 0.0 [0.0-3.8] vs 102.3 [19.8-267.5]; *P =* 0.034) (Figure 2A), suggesting a greater antiproliferative effect with the dual API DCB than with the PTX DCB. A similar trend was observed for the number of BrdU-positive nuclei in the intima (median [Q1-Q3]: 0.0 [0.0-0.8] vs 62.0 [8.2-109.3]; *P =* 0.034) (Figure 2B). Comparing the percentage of BrdU-positive nuclei in the intima between the 2 groups, the median value of percent BrdU-positive nuclei in the dual API DCB was significantly lower than in the PTX DCB (median [Q1-Q3]: 0.0% [0.0%-3.6%] vs. 30.3% [12.3%-63.5%]; *P =* 0.034) (Figure 2C). A similar assessment for the media showed a statistical trend toward a greater percentage of BrdU-positive nuclei in the dual API DCB than in the PTX DCB (median [Q1-Q3]: 24.3 [20.9-26.7] vs 18.7 [9.8-23.2]; *P =* 0.092) (Figure 2F), whereas there was no statistical difference between the dual API DCB and the PTX DCB in the number of nuclei (median [Q1-Q3]: 1,095.3 [790.7-1285.2] vs 1,061.7 [596.0-1256.2]; *P =* 0.67) (Figure 2D) and the number of BrdU-positive nuclei (median [Q1-Q3]: 238.0 [187.8-312.2] vs 221.3 [63.7-241.7]; *P =* 0.35) (Figure 2E). These results suggest that the dual API DCB had a greater antiproliferative effect in the intima rather than in the media compared to the PTX DCB at 5 days after the treatment. Representative histology sections of the vessels treated with the 2 devices after 5 days are shown in Figure 3.

#### Vascular response at 28 days

**Figure 4:**
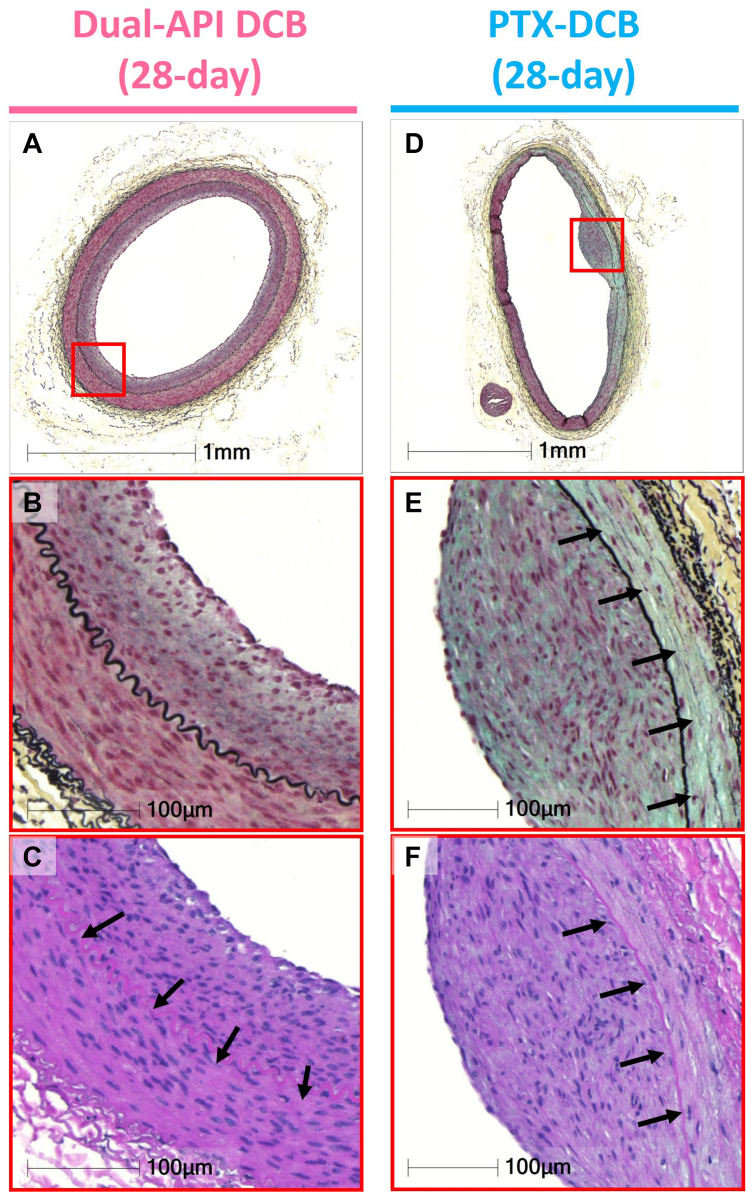
Representative Histology of Rabbit Iliac Arteries 28 Days After Treatment With Dual API or PTX DCB Histology images of the iliac artery treated with (A to C) the dual API DCB and (D to F) the PTX DCB. Movat pentachrome (MP)-stained section of an iliac artery treated with (A) the dual API DCB or (D) the PTX DCB. A uniform neointima was seen circumferentially in the artery treated with the dual API DCB, whereas a nonuniform neointimal formation was observed in the artery treated with the PTX DCB. The boxed areas in A and D are shown at high-power images with (B and E) MP and (C and E) hematoxylin and eosin. Neointimal formation was minimally observed without the loss of SMC in the media of the artery treated with the (C) dual API DCB (arrows), whereas mild neointimal formation was observed with deposition of (E) the extracellular matrix (arrows) and (F) the loss of SMC (arrows) in the medial of the artery treated with the PTX DCB. Abbreviations as in Figures 1 and 3.

The subacute vascular response was performed histologically on 4 arteries treated with the dual API DCB and 4 arteries treated with the PTX DCB from the 4 animals sacrificed at 28 days. Histomorphometry showed comparable percent stenosis between the arteries treated with the dual API DCB or the PTX DCB (median [Q1-Q3]: 23.9% [19.2%-35.9%] vs 19.3% [14.8%-22.4%]; *P =* 0.20) in addition to other morphometric parameters, which also showed no significant differences between the 2 groups (Table 2). In the semiquantitative scores for each histologic parameter, the dual API DCB showed significantly less smooth muscle cell (SMC) loss than the PTX DCB in both depth (median [Q1-Q3]: 0.0 [0.0-0.0] vs 2.5 [0.4-3.8]; *P =* 0.047) (Table 2) and circumference (median [Q1-Q3]: 0.0 [0.0-0.0] vs 1.3 [0.2-2.0]; *P =* 0.045) (Table 2). Although other parameters were not significantly different between the 2 groups, the dual API DCB group had a statistical trend toward lower PG/collagen scores compared to the PTX-DCB group (median [Q1-Q3]: 0.0 [0.0-0.5] vs 1.5 [0.3-2.2]; *P =* 0.093) (Table 2). These results represent lower injury of the media in the dual API DCB compared to the PTX DCB at 28 days. Representative histology sections of the vessels treated with the 2 devices after 28 days are shown in Figure 4.

### Coronary and downstream myocardial effects at 28 days in the porcine model

There was no significant difference in the quantitative coronary angiography (QCA) parameters including percent diameter stenosis between the dual API DCB, PTX DCB, and POBA groups at baseline and the 28-day follow-up with the exception of the mean lumen diameter post-treatment at baseline (median [Q1-Q3]: 2.1 [2.0-2.30] mm vs 1.8 [1.5-2.4] mm vs 2.8 [2.6-3.1] mm; *P =* 0.021) and the mean lumen diameter at the 28-day follow-up (median [Q1-Q3]: 2.5 [2.3-2.7] mm vs 2.4 [2.3-2.6] mm vs 3.0 [2.8-3.3] mm; *P =* 0.035) (Supplemental Table 4). In the analysis of treated coronary artery segments, vessel morphometry (ie, histologic analysis) showed a significant difference among the dual API DCB group, the PTX group, and the POBA group in the external elastic lamina (EEL) area (median [Q1-Q3]: 4.05 [3.9-4.4] mm^2^ vs 4.4 [4.0-5.4] mm^2^ vs 5.7 [4.8-6.9] mm^2^; *P =* 0.039), internal elastic lamina (IEL) area (median [Q1-Q3]: 3.4 [3.1-3.7] mm^2^ vs 3.6 [3.1-4.5] mm^2^ vs 4.7 [4.0-5.7], mm^2^; *P =* 0.040), lumen area (median [Q1-Q3]: 3.2 [2.8-3.5] mm^2^ vs 3.3 [2.9-4.4] mm^2^ vs 4.6 [4.0-5.7] mm^2^; *P =* 0.028), and mean intimal thickening (median [Q1-Q3]: 0.06 [0.04-0.07] mm vs 0.04 [0.03-0.08] mm vs 0.01 [0.0002-0.02] mm; *P =* 0.035) (Supplemental Table 5). These differences are thought to be caused by the fact that POBA was used in the right coronary artery in every animal, which was an anatomically larger vessel than the left coronary arteries (ie, the left anterior descending and left circumflex arteries) that were treated with the dual API DCB or the PTX DCB. Histologic scoring for the PG/collagen score in the media was the greatest for the PTX DCB group compared to the dual API DCB and POBA groups (median [Q1-Q3]: 2.0 [1.8-2.3] vs 1.0 [0.8-1.5] vs 1.0 [0.8-1.3]; *P =* 0.026) (Supplemental Table 5). Histologic findings at 28 days in the porcine coronary model showed consistent results with the rabbit iliac model in terms of less medial injury with the dual API DCB compared to the PTX DCB.

**Figure 5:**
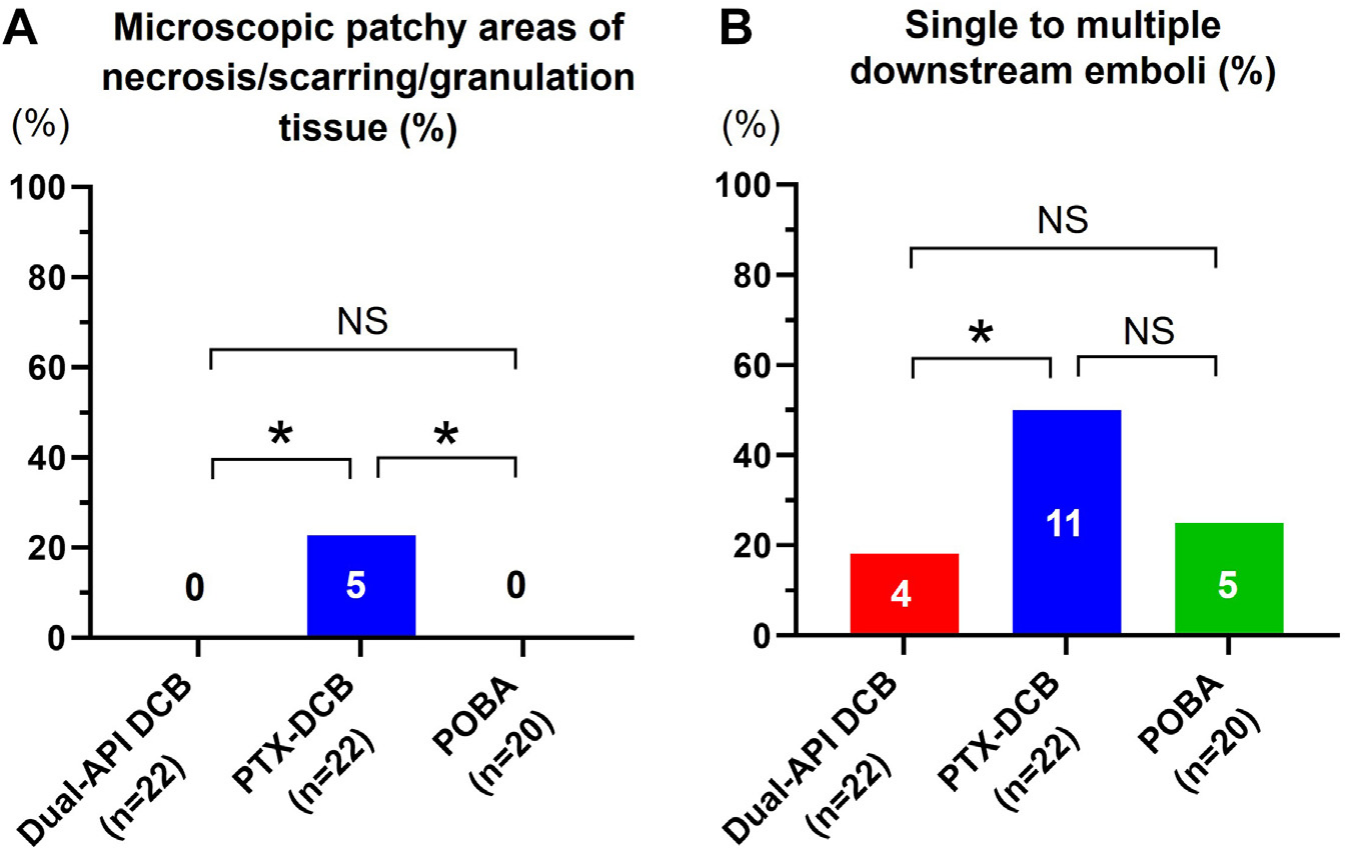
Comparison of Findings From Downstream Myocardium 28 Days After Dual API, PTX DCB, or POBA Treatment (A) The percentage of histology sections with necrosis, scarring, or granulation. (B) The percentage of histology sections with single to multiple downstream emboli. Data are presented from 4 animals total who received treatment with the dual API DCB, PTX DCB, or plain old balloon angioplasty (POBA) in 3 coronary arteries with respective downstream myocardial territories sampled for histology. All statistical analyses were performed using the chi-square test. ∗*P <* 0.05. Abbreviations as in Figure 1.

**Figure 6:**
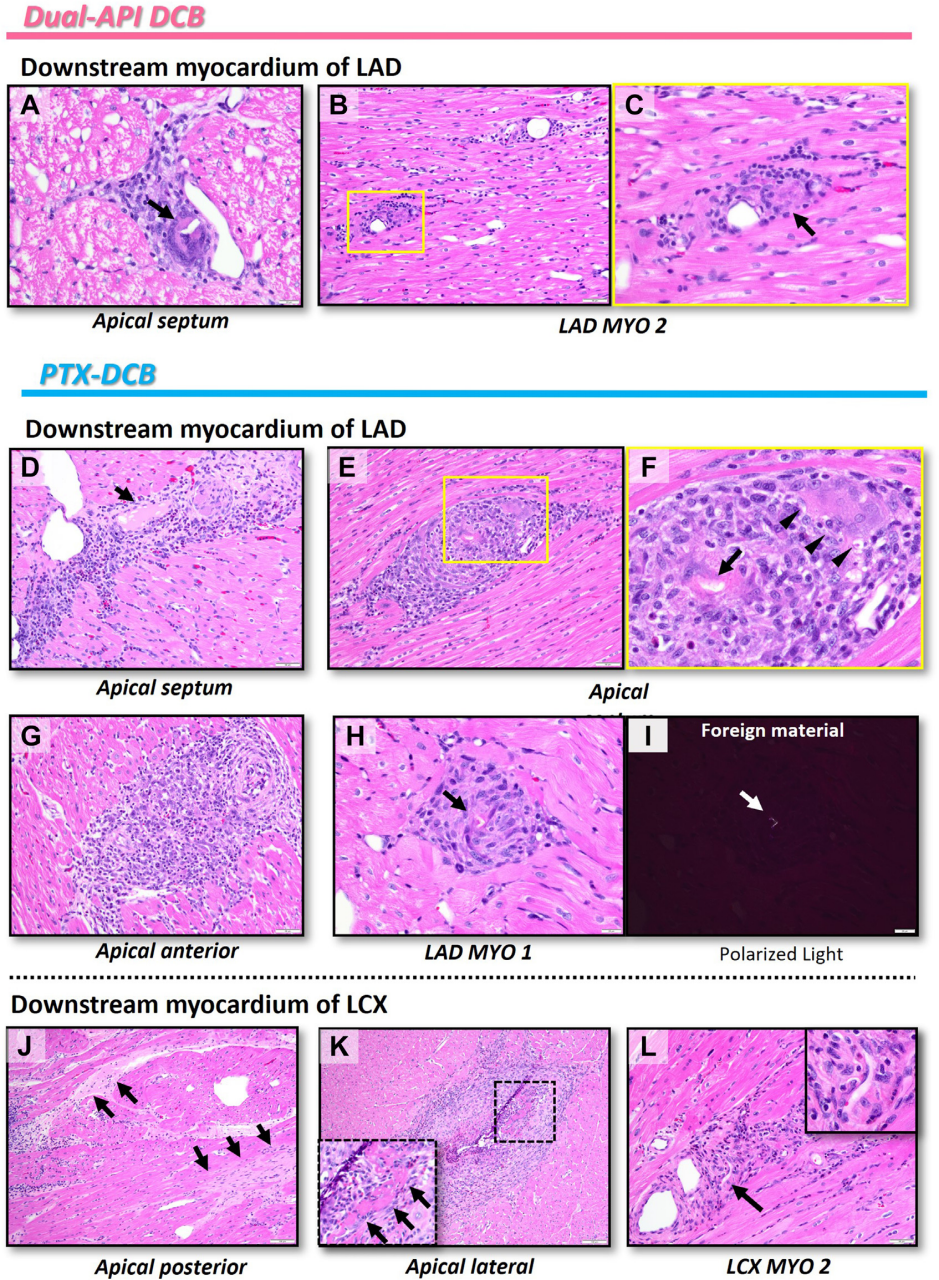
Representative Histology of Downstream Myocardium at 28 Days From Healthy Swine After Treatment With Dual API DCB or PTX DCB (A to C) Histology demonstrated no findings of tissue injury and few regions with foreign material in the downstream myocardial regions from the animal treated with the dual API DCB, whereas (D to L) multiple regions showed tissue injury and presence of foreign material in the downstream myocardium in the regions from the animal treated with the PTX DCB. All rectangles in the figure are shown as high-power images in the same panel or the next panel. (A) The region of the apical septum showed foreign material embedded in luminal fibrin with prominent giant cells (arrow). (B and C) The section of the left anterior descending (LAD) myocardium 2 (MYO 2) showed perivascular inflammation, including (C) giant cells (arrow). (D to L) Histology of the downstream myocardium after treatment with the PTX DCB. (D to F) The section of the apical septum shows granulation tissue with (D) microscopic necrosis (arrow) and (E and F) foreign material (arrow) with surrounding inflammation including giant cells (arrowheads). (G) The region of the apical anterior myocardium shows severe perivascular inflammation with giant cells. (H and I) The LAD MYO 1 showed the presence of (H) foreign material (arrow) with a (I) bright area by polarized light (arrow) with surrounding inflammation. (J to L) The downstream myocardium of the left circumflex (LCX) treated with the PTX DCB showed necrosis in the (J) apical posterior (arrows) and (K) the apical lateral (arrows). (L) Foreign material with surrounding inflammation was observed in the section of LCX MYO 2 (arrow). Abbreviation as in Figure 1.

We next evaluated the downstream myocardium of treated coronary segments. A total of 64 tissue sections from 12 regions were evaluated with 22 sections from 4 regions from the dual API and PTX DCB treatment groups, respectively, as well as 20 sections from 4 regions from the POBA treatment group (Supplemental Table 6). There was no evidence of tissue injury, defined as necrosis, scarring, and granulation in the downstream myocardium regions of coronary arteries treated with the dual API DCB and POBA, whereas downstream myocardium regions in 3 of the 4 coronary arteries treated with the PTX DCB showed tissue injury (Supplemental Table 7). For the section-based analysis, 0 of 22 and 0 of 20 sections from the myocardium downstream of the coronary arteries treated with the dual API DCB and the POBA group showed evidence of tissue injury, whereas 5 of 22 of sections in the PTX DCB group demonstrated tissue injury (0% vs 0% vs 23%) (Figure 5A), a difference that was significant between the dual API DCB group and the PTX group (*P =* 0.018) as well as between the POBA group and the PTX group (*P =* 0.023). We also examined the presence of distal emboli in the downstream myocardium. Emboli were observed in 2 of the 4 regions of the downstream myocardium treated with the dual API DCB as well as the POBA group, whereas 3 of the 4 regions of the downstream myocardium treated with the PTX DCB showed emboli (Supplemental Table 8). Section-based analysis showed the least number of sections showing downstream emboli in the dual API DCB compared to the POBA and PTX-DCB groups (4/22 [18%] vs 5/20 [25%] vs 11/22 [50%]) (Figure 5B) (dual API group vs PTX group: *P =* 0.026 and POBA group and PTX DCB group: *P =* 0.096). These findings indicate that treatment with the dual API DCB and POBA led to no myocardial injury and less downstream emboli compared to the PTX DCB. Representative histology images of the downstream myocardial areas from coronary vessels treated with the dual API DCB or the PTX DCB are shown in Figure 6.

## Discussion

To the best of our knowledge, no study has investigated the combination use of a PTX and SRL DCB or the sequential use of these 2 types of DCBs in the clinical settings or in animal models for vascular proliferative disorders.^18^ In the present study, the efficacy of the combination of PTX and SRL within dual API NPs was evaluated for the first time in both cell culture and preclinical experiments. The main findings of our studies with combination treatment in NPs were: 1) the dominant mechanism of the inhibition of cell proliferation with the dual API NPs is via cell cycle arrest with the suggestion that synergy between SRL and PTX produces a strong antiproliferative effect without excessive cell toxicity that is seen with PTX alone; 2) the pharmacokinetic assessment demonstrated a similar ratio of concentrations of SLR and PTX in arterial tissue at 28 days after treatment as coated on the dual API DCB; 3) the dual API DCB significantly inhibited intimal cell proliferation in the acute phase (5 days) compared to the PTX DCB; 4) at 28 days, the dual API DCB showed lower injury of the media compared to the PTX DCB; and 5) treatment with the dual API DCB in coronary arteries caused no myocardial injury, whereas treatment with the PTX DCB caused significantly high myocardial injury and embolism.

### Mechanism of inhibition of cell proliferation with dual API NPs

Although SRL and PTX in combination have been tested for cancer therapy to induce greater cytotoxic effects on cancer cells than individual agents alone,^19^, ^20^, ^21^, ^22^ the 2 combined drugs remained unexplored for treating vascular diseases. This drug combination strategy is used to explore the synergistic effects of the combination, which is defined as when their combined effect is greater than that predicted by their individual potencies.^23^ The synergistic effect is attributed to the inhibition of multiple signaling pathways and receptors involved in cell proliferation.^24^ Although the specific molecular mechanism of the inhibition of VSMCs needs further investigation, proliferating VSMCs also share similar pathways and oncogenes in cell proliferation, such as the mammalian target of rapamycin signaling pathway in cell proliferation.^25^ In our study, we achieved the synergistic effect of the combination while keeping the predominant mechanism of the inhibition of cell proliferation via cell cycle arrest rather than the cytotoxic effect of PTX. With dual API NPs, the same degree of inhibition of cell proliferation (IC_50_) was achieved via cell cycle arrest with an ∼15-fold lower SRL dose than SRL alone or at a 50% lower PTX dose than PTX alone (Figure 1). In fact, the SRL dose used in the combination has no significant effect on the inhibition of cell proliferation when used alone. The safety of SRL is considered to be caused by its cytostatic mechanism of action, unlike that of PTX, which is via the cytotoxic effect that can have an effect on the target artery (eg, aneurysm caused by the loss of VSMCs).^26^^,^^27^

### Neointimal formation after dual API DCB treatment

The dual API DCB is a novel device that adapts the right combination profile of PTX and SRL (1:9 w/w, PTX:SRL), considering both efficacy and safety in the field of vascular disease. Both the drugs were encapsulated into the same NPs to ensure that they are delivered to the target vessel in the same synergistic combination ratio. In addition, the coating was developed so that there is no loss of drug during the handling of the balloon, which is a significant concern with some of the currently used PTX DCBs that show a loss of 20% to 40% of the coated drug.^28^ In the rabbit iliac artery model, in the acute phase (5 days), the dual API DCB showed significant inhibition of cell proliferation as evidenced by less expression of BrdU-positive nuclei than with the conventional PTX DCB (Figure 2). In the subacute phase (28 days), the inhibitory effect of the dual API DCB on intimal proliferation remained comparable to that of the PTX DCB. A long-term study (>90 days) would further confirm the antiproliferative effect of the dual API DCB.

#### Vascular healing after the dual API DCB treatment

The medial injury, which is evidenced by the loss of smooth muscle cells, was significantly less with the dual API DCB in the subacute phase at 28 days compared to the PTX DCB. The greater expression of BrdU in media in the dual API DCB group compared to the PTX DCB group during the acute phase at 5 days after treatment suggests that there was cell proliferation and, thus, a healing response in the injured media. These results support the finding that medial injury was less in the dual API DCB compared to the PTX DCB in the subacute phase (28 days). Based on these results, the effect of the dual API DCB appears to be more physiologic vascular healing, with the drugs inhibiting cell proliferation primarily in the intima to prevent restenosis while minimizing the effects of the drugs on smooth muscle cells in the injured media. This effect can be attributed to the cell cycle arrest mechanism of the dual API DCB than the cytotoxic effect of the PTX DCB.

### Distal embolism after DCB treatment

PTX can be classified into 2 main formulations based on their different morphologies: crystalline and amorphous formulations. Crystalline formulations, which are composed of fine crystals, are relatively stable and can provide controlled release characteristics. On the other hand, amorphous formulations exist in an uncrystallized form and are more soluble than crystalline formulations, resulting in faster drug release.^29^ In the development of the DCB, the excipients/carriers used to deliver the drug, the crystallinity of the drug, and the coating technology used are very important, and these materials determine the trade-off between its efficacy and the occurrence of distal emboli. Preclinical animal studies have reported histologic evidence that various features of the drug coating on DCBs (ie, drug formulation, dosage, and excipients) can develop embolization of the dislodged foreign material in the downstream tissue.^3^^,^^5^ Furthermore, the currently used DCBs carry PTX drug crystals as large as 300 μm,^30^ which are significantly larger than the diameter of downstream arteriole (74-85 μm).^12^^,^^31^ In our study, the assessment of myocardial tissue downstream of the treated coronary arteries showed an improved safety profile, with significantly less distal embolization of the downstream myocardium and no tissue damage with the dual API DCB compared to the single PTX DCB. It is still controversial if the distal emboli after the DCB treatment affect clinical outcomes.^32^, ^33^, ^34^, ^35^, ^36^, ^37^ However, it is difficult to identify the existence of a distal embolus itself directly, even with the currently available imaging modalities such as computed tomography, because of the small size of the embolus material, which is less than the imaging resolution (500 μm). Therefore, given the lack of robust evidence that emboli themselves determine the clinical prognosis, further development of DCB techniques with a reduced risk of downstream embolic effects is warranted.

### Study limitations

We used a relatively small sample size in these studies. The drug concentration was also not assessed for all of the arteries treated with DCBs. The comparator PTX DCB devices were different in experiments 2 and 3, and the degree of overstretch used when deploying DCBs in each model was also not the same. For 2 types of control PTX DCBs, there are still no randomized controlled trials reporting on their efficacy and safety for the treatment of de novo disease, although a randomized study using the agent DCB for in-stent restenosis is ongoing and is expected to be published soon.^38^ The data were evaluated at the 28-day follow-up, and further evaluation of the vascular response as well as the downstream myocardium extended time points during the chronic phase is needed. PTX NPs or SRL NPs alone at the same concentrations as those loaded on the dual API DCB were not evaluated in these animal studies.

## Conclusions

A novel dual-agent combination of PTX and SRL coincorporated into NPs demonstrated synergistic effects for use in DCBs by inhibiting cell proliferation via cell cycle arrest rather than cytotoxicity. In a rabbit iliac artery model, the dual API DCB demonstrated better inhibition of neointimal proliferation in the acute phase compared to the conventional PTX DCB, supporting the idea that the 2 drugs are effective in the acute phase of DCBs. Furthermore, the better healing of medial injury in the subacute phase and the lower incidence of tissue damage and distal emboli in the downstream myocardium in the porcine model highlight the greater efficacy and safety of the dual API DCB over the conventional PTX DCB. Further investigation of the efficacy of the dual API DCB in humans is warranted.

Perspectives **COMPETENCY IN MEDICAL KNOWLEDGE:** Both efficacy and safety of DCBs are crucial considerations. The dual API DCB, which combines SRL and PTX in sustained release, biodegradable nanoparticles, successfully fulfills these requirements. Preclinical data demonstrate improved outcomes in terms of cell proliferation inhibition during the acute phase and vascular healing during the chronic phase.

**TRANSLATIONAL OUTLOOK:** The combination treatment of SRL and PTX achieves these effects by using cell cycle arrest as the mechanism of inhibition, distinguishing it from the cytotoxic action of PTX. Dual API DCBs take advantage of the antiproliferative property of both the drugs while minimizing toxicity. In addition to pharmacologic superiority, the dual API DCB significantly reduces downstream embolization and myocardial injury compared to the commercially available PTX DCBs. Furthermore, it avoids the issue of coating flaking, a common concern with the current DCBs. SirPlux Duo, or the dual API DCB, offers superior features compared to conventional PTX DCBs.

## Funding Support and Author Disclosures

This study was funded by Advanced NanoTherapies, Inc. CVPath Institute has received institutional research support from R01 HL141425, RECOVER Initiative (OT2HL161847-01), National Institutes of Health RECOVER480 (OT2HL161847-01, PATHO-PH1-SUB_04_22), Biomedical, 4C Medical, 4Tech, Abbott Vascular, Ablative Solutions, Absorption Systems, Advanced NanoTherapies Inc, Aerwave Medical, Alivas, Amgen, Asahi Medical, Aurios Medical, Avantec Vascular, BD, Biosensors, Biotronik, Biotyx Medical, Bolt Medical, Boston Scientific, Canon, Cardiac Implants, Cardiawave, CardioMech, Cardionomic, Celonova, Cerus EndoVascular, Chansu Vascular Technologies, Children’s National, Concept Medical, Cook Medical, Cooper Health, Cormaze, CRL, Croivalve, CSI, Dexcom, Edwards Lifesciences, Elucid Bioimaging, eLum Technologies, Emboline, Endotronix, Envision, Filterlex, Imperative Care, Innovalve, Innovative, Cardiovascular Solutions, Intact Vascular, Interface Biolgics, Intershunt Technologies, Invatin, Lahav, Limflow, L and J Bio, Lutonix, Lyra Therapeutics, Mayo Clinic, Maywell, MDS, MedAlliance, Medanex, Medtronic, Mercator, Microport, Microvention, Neovasc, Nephronyx, Nova Vascular, Nyra Medical, Occultech, Olympus, Ohio Health, OrbusNeich, Ossiso, Phenox, Pi-Cardia, Polares Medical, Polyvascular, Profusa, ProKidney, Protembis, Pulse Biosciences, Qool Therapeutics, Recombinetics, Recor Medical, Regencor, Renata Medical, Restore Medical, Ripple Therapeutics, Rush University, Sanofi, Shockwave, SMT, SoundPipe, Spartan Micro, Spectrawave, Surmodics, Terumo Corporation, The Jacobs Institute, Transmural Systems, Transverse Medical, TruLeaf, UCSF, UPMC, Vascudyne, Vesper, Vetex Medical, Whiteswell, WL Gore, and Xeltis. Mr Nowicki is an employee of Advanced NanoTherapies, Inc. Dr Labhasetwar is the inventor of dual API nanotechnology (Cleveland Clinic licensed the technology to Advanced NanoTherapies, Inc); serves as a consultant for Advanced NanoTherapies; and has equity in Advanced NanoTherapies, Inc; his conflict of interest is managed by the Conflict-of-Interest Committee of the Cleveland Clinic. Dr Finn has received honoraria from Abbott Vascular, Biosensors, Boston Scientific, Celonova, Cook Medical, CSI, Lutonix Bard, Sinomed, and Terumo Corporation; and is a consultant to Amgen, Abbott Vascular, Boston Scientific, Celonova, Cook Medical, Lutonix Bard, and Sinomed. All other authors have reported that they have no relationships relevant to the contents of this paper to disclose.
