# Supplementary material for: Efficacy and Safety of Dual Paclitaxel and Sirolimus Nanoparticle-Coated Balloon
Source: JACC Basic Transl Sci. 2024 May 1;9(6):774–89. doi: 10.1016/j.jacbts.2024.02.002 (PMC11282887; doi:10.1016/j.jacbts.2024.02.002)
Supplement: Supplemental Material [file mmc1.pdf]

Supplemental Material

Efficacy and Safety of Dual Paclitaxel and Sirolimus Nanoparticle Coated Balloon

Author list:
Kenji Kawai, MD[1]; Mohammed Tanjimur Rahman, PhD[2]; Ryan Nowicki, BS[3]; Frank D. Kolodgie, PhD[1]; Atsushi Sakamoto, MD[1]; Rika Kawakami, MD[1]; Takao Konishi, MD[1]; Renu Virmani, MD[1]; Vinod Labhasetwar, PhD[2*]; Aloke V. Finn, MD[1,4*]

Affiliations:
1. CVPath Institute, Gaithersburg, MD, USA.
2. Department of Biomedical Engineering, Lerner Research Institute, Cleveland Clinic, Cleveland, OH 44195, USA
3. Advanced NanoTherapies, Inc., Los Gatos, CA, USA
4. University of Maryland, School of Medicine, Baltimore, MD, USA

Corresponding authors:
*Aloke V. Finn (Primary)
19 Firstfield Road, MD, 20878
E-mail: afinn@cvpath.org
Tel: 301.208.3570
Fax: 301.208.3745

*Vinod Labhasetwar, Ph.D.
Biomedical Engineering, Lerner Research Institute
Cleveland Clinic, Cleveland, OH 44195
E-mail: labhasv@ccf.org
Tel: 216-445-9364

Material:

Supplemental Methods
8 Supplemental Tables
1 Supplemental Figures

Supplemental Methods

In vitro cell culture studies

In the initial set of experiments, keeping the SRL concentration constant in solution (100 or 1000 ng/ml), the cells were treated with different doses of PTX in solution to determine the most effective synergistic drug combination. The subsequent experiment was carried out as above with the encapsulated PTX and SRL and finally with co-encapsulated PTX and SRL nanoparticle formulation. The inhibition of cell proliferation was determined using the CyQUANT™ NF Cell Proliferation Assay kit (Life Technologies, Carlsbad, CA). The IC_{50} for each treatment was calculated using the non-linear curve fit (logistic) using OriginPro 8 (OriginLab Corp., Northampton, MA). The above series of experiments determined that the 1:9 w/w ratio of PTX: SRL is highly synergistic; hence, the above ratio of the two drugs co-encapsulated in NPs was further investigated, particularly to understand the mechanism of inhibition of cell proliferation. It was demonstrated that encapsulation enhances the antiproliferative effect of the combination treatment as compared to drugs in solution in the same combination ratio.

Analyzing dead/live cells and BrdU positive proliferating cells in cell culture experiment

NPs without any encapsulated API acted as a control group. The IC_{50} for each treatment was determined in HVSM cells (SRL=3,100 ng/mL, PTX = 45 ng/mL, and combination = 225 ng/mL). Four hours prior to 3 day incubation period, the cells were imaged using an EVOS XL Core phase contrast microscope (Advanced Microscopy Group, Bothell, WA). From these images, total cells and apoptotic/dead cells were counted from ten random fields out of three replicate wells, and the results were plotted as a percentage of apoptotic cells with respect to the total number of cells.

Immediately after imaging, the cells were incubated with BrdU (5-bromo-2'-deoxyuridine), Thymidine analog (Cat# ab142567, Abcam, Cambridge, MA) dissolved in cell culture media at 10 µM concentration for four hours to determine proliferating cells. The cell culture medium was removed from the well, and the cells were fixed with 4% paraformaldehyde. The cells following immunocytochemical staining with eBioscience BrdU kit (Cat# 8800-6599, ThermoFisher Scientific) and counterstaining with hematoxylin were imaged using Aperio AT2 DX microscope (Leica Biosystems, Wetzler, Germany). Anti-BrdU stained proliferative positive cells were identified and counted manually from ten randomly selected areas of the images using Aperio ImageScope software (Leica Biosystems)

Tissue processing and histology preparation in rabbit iliac model experiment

Immediately after euthanasia, the treated iliofemoral arteries were carefully dissected free from surrounding tissue and flushed with 50-100 ml of heparinized lactate ringers to remove residual blood. All the tissue was treated in stages with alcohol and xylene. After dehydration, each sample was further sectioned into 3-4 mm intervals and sequentially placed in a block, keeping the longitudinal orientation. Tissue samples were cut into 5-6 µm sections with a rotary microtome, mounted on charged slides, and stained with hematoxylin and eosin (H&E) and modified Movat Pentachrome (MP) stains. For the immunohistochemical evaluation, the primary antibody for BrdU (cat#MO744, DAKO/Agilent, Santa Clara, CA) was used at a concentration of 1:200. The effect of BrdU was confirmed by sections of the small intestine of each animal (internal positive control).

Morphometry and histology analysis in rabbit iliac model experiment

Quantitative assessments were performed to determine the morphologic effects of treatment. MP-stained histological sections were analyzed using digital planimetry with a microscope system calibrated to perform the measurements [NIST Trackable Calibrated Microscope System (IP Lab Software, Rockville, MD)]. Subsequently, to assess arterial injury and healing, histologic parameters were examined for endothelial loss, platelets/fibrin, surface and medial, medial extracellular matrix (ECM)/collagen, calcification, intimal/medial and adventitial inflammation, medial and adventitial red blood cells (bleeding), medial SMC loss and medial injury.

Coronary artery catheterization and intervention in swine heart model

At the initial procedure on day 0, angiography was performed with contrast medium after the administration of nitroglycerin (100 µg), then all the devices were delivered over a 0.014-inch guidewire to the target vessels. Endothelial denudation was performed at targeted sites using a plain uncoated balloon at the selected sites prior to treatment with DCB. Balloon expansion pressure for the denudation was based on the online Quantitative coronary angiography (QCA) diameter and the provided balloon compliance chart. Dual-API DCB or PTX-DCB 3.0 x 20 mm were then deployed over a guidewire at the target site in the LAD or LCX, and POBA 3.0 x 20 mm was deployed in the RCA. Each balloon was inflated for 60 seconds with a target balloon-to-vessel ratio of at least 10% based on a compliance chart that achieved a range of 0.99:1 to 1.3:1. Overall, both the LAD and LCX of each animal were treated with either Dual-API DCB or PTX-DCB, and the RCA of each animal was treated with POBA.

Termination of swine model experiment

Immediately after termination, the heart was dissected from the surrounding tissue, carefully excised, flushed with 500-1000 mL of heparinized lactate Ringer's solution under 80-100 mmHg pressure to remove residual blood, and gravity perfusion fixed in 10% neutral buffered formalin (NBF) for 20 minutes. The heart samples were immersion-fixed overnight in 10% NBF and then transported to CVPath Institute for histological processing and analysis.

Supplemental Table 1. Description of drug coated balloons used in each experiment.

	SirPlux Duo™	PREVAIL™	AGENT™
Manufacturer	Advanced Nanotherapies	Medtronic	Boston Scientific
Dual or single drug	Dual	Single	Single
Drug	Paclitaxel and Sirolimus	Paclitaxel	Paclitaxel
Excipient	f-NPs	PowerTrac™	Trans Pax™
Drug dose ($\mu g/mm^2$)	1.5*	3.5	2.0
Used in animal experiment	Experiment 2 and 3	Experiment 2	Experiment 3

*1.5 $\mu g/mm^2$ is consisted of SRL 1.35 $\mu g/mm^2$ and PTX 0.15 $\mu g/mm^2$

Supplemental Table 2. The areas of downstream myocardium corresponding to the treated coronary artery

Coronary Artery	LAD	LCX	RCA
The region of downstream Myocardium	Apical anterior<br>Mid anterior,<br>Apical septum<br>Mid septum<br>LAD MYO 1<br>LAD MYO 2	Apical lateral<br>Mid lateral<br>Apical posterior<br>LCX MYO 1<br>LCX MYO 2	Apical RV<br>Mid RV<br>Mid Posterior<br>RCA MYO 1<br>RCA MYO 2

MYO 1 and MYO 2 are two areas near the treated coronary artery. LAD= left anterior descending artery, LCX= left circumflex artery, RCA = right coronary artery, RV = right ventricle

Supplemental Table 3. Percentage of cells in different phases of cell-cycle

**Cell-Cycle**	0 hour	After 3 days of incubation		
	Untreated	SRL-NPs	PTX-NPs	Dual API-NPs
**G0/G1**	47.4%	77.1%	45.8%	80.1%
**S**	42.4%	16.9%	19.1%	8.8%
**G2/M**	4.7%	5.3%	31.5%	10.2%
**Apop**	3.3%	0.6%	3.6%	0.9%

Supplemental Table 4. Angiographic measurements at baseline and 28 days follow-up in porcine coronary model.

Phase	Diameter	Dual-API DCB (n=4)	PTX DCB (n=4)	POBA (n=4)	P-value
Pre-treatment	Mean LD (mm)	2.7 [2.6-2.8]	2.7 [2.6-2.9]	3.0 [2.8-3.1]	0.087
Balloon inflation	Balloon to Artery Ratio	1.1 [1.0-1.1]	1.0 [1.0-1.0]	1.0 [1.0-1.1]	0.061
Post-treatment	Mean LD (mm)	2.1 [2.0-2.3]	1.8 [1.5-2.4]	2.8 [2.6-3.1]	0.021
28-days follow up	Mean LD (mm)	2.5 [2.3-2.7]	2.4 [2.3-2.6]	3.0 [2.8-3.3]	0.035
	% diameter stenosis (%)	25.5 [22.5-27.8]	25.3 [18.2-34.6]	17.6 [14.6-19.3]	0.084

LD, lumen diameter; Values are presented as median [25th-75th percentiles].

Supplemental Table 5. Vessel morphometry and histologic results at 28 days in porcine coronary model

	**Agent DCB (n=4)**	**Dual-API (n=4)**	**POBA (n=4)**	**P-value**
Morphometry				
EEL Area (mm2)	4.1 [3.9-4.4]	4.4 [4.0-5.4]	5.7 [4.8-6.9]	0.039
IEL Area (mm2)	3.4 [3.1-3.7]	3.6 [3.1-4.5]	4.7 [4.0-5.7]	0.040
Lumen Area (mm2)	3.2 [2.8-3.5]	3.3 [2.9-4.4]	4.6 [4.0-5.7]	0.028
Medal Area (mm2)	0.7 [0.7-0.8]	0.8 [0.8-1.0]	1.0 [0.8-1.5]	0.14
Neointimal Area (mm2)	0.2 [0.1-0.3]	0.1 [0.1-0.3]	0.03 [0.01-0.06]	0.08
% Stenosis	5.5 [4.3-8.4]	2.7 [1.5-9.8]	0.8 [0.2-1.1]	0.09
THK mean	0.06 [0.04-0.07]	0.04 [0.03-0.08]	0.01 [0.0002-0.02]	0.035
Histology parameters				
EC Loss Score	0.2 [0.0-0.6]	0.0 [0.0-0.3]	0.3 [0.0-0.7]	0.55
Fibrin/Platelet Thrombus (Surface)	0.0 [0.0-0.0]	0.0 [0.0-0.0]	0.0 [0.0-0.0]	1.00
Medial/Neointimal fibrin	0.2 [0.0-0.3]	0.0 [0.0-1.0]	0.0 [0.0-0.0]	0.36
Proteoglycan/collagen (Media)	2.0 [1.8-2.3]	1.0 [0.8-1.5]	1.0 [0.8-1.3]	0.026
Inflammation (Intima or Media)	0.0 [0.0-0.0]	0.0 [0.0-0.0]	0.0 [0.0-0.0]	1.00
Calcification	0.0 [0.0-0.0]	0.0 [0.0-0.0]	0.0 [0.0-0.0]	1.00
Medial SMC Loss (Depth)	0.7 [0.1-1.8]	0.7 [0.2-1.9]	0.0 [0.0-0.3]	0.18
Medial SMC Loss (Circumference)	0.7 [0.1-1.0]	0.33 [0.1-1.1]	0.0 [0.0-0.3]	0.23

All continuous values are expressed with median [25th-75th percentiles]. Adv = adventitia, EC = endothelial cell, SMC = smooth muscle cells, THK = thickness of neointima

Supplemental Table 6. Number of assessed sections of downstream myocardium in each animal

	LAD	LCX	RCA
Animal 1	Dual API	Dual API	POBA
	6	5	5
Animal 2	PTX-DCB	PTX-DCB	POBA
	6	5	5
Animal 3	PTX-DCB	PTX-DCB	POBA
	6	5	5
Animal 4	Dual API	Dual API	POBA
	6	5	5

Supplemental Table 7. Sections of downstream myocardium with tissue injury detected by histology.

	Myocardium region treated with	LAD	LCX	RCA
Animal 1	Treatment group	Dual API	Dual API	POBA
	Region of sections with tissue injury	No findings	No findings	No findings
	Number of sections with tissue injury	0 of 6	0 of 5	0 of 5
Animal 2	Treatment group	PTX-DCB	PTX-DCB	POBA
	Region of sections with tissue injury	AP, AS	AL, AP	No findings
	Number of sections with tissue injury	2 of 6	2 of 5	0 of 5
Animal 3	Treatment group	PTX-DCB	PTX-DCB	POBA
	Region of sections with tissue injury	AA	No findings	No findings
	Number of sections with tissue injury	1 of 6	0 of 5	0 of 5
Animal 4	Treatment group	Dual API	Dual API	POBA
	Region of sections with tissue injury	No findings	No findings	No findings
	Number of sections with tissue injury	0 of 6	0 of 5	0 of 5

AA = apical anterior, AL = apical lateral, AP = apical posterior, AS = apical septum, MA = mid anterior, ML = mid lateral, MS = mid septum, MP = mid posterior, LAD = left anterior descending artery, LCX = left circumflex artery, RCA = right coronary artery, RV = right ventricle.

Supplemental Table 8. Sections of downstream myocardium with emboli detected by histology.

	Myocardium region treated with	LAD	LCX	RCA
Animal 1	Treatment group	Dual API	Dual API	POBA
	Region of sections with emboli	AS, LAD MYO 2	No findings	No findings
	Number of sections with emboli	3 of 6	0 of 5	0 of 5
Animal 2	Treatment group	PTX-DCB	PTX-DCB	POBA
	Region of sections with emboli	AA, MA, AS, MS, LAD MYO 1	AL, ML, AP, LCX MYO 1, LCX MYO 2	Mid RV, MP, RCA MYO 1 RCA MYO 2
	Number of sections with emboli	5 of 6	5 of 5	4 of 5
Animal 3	Treatment group	PTX-DCB	PTX-DCB	POBA
	Region of sections with emboli	No findings	LCX MYO 2	MP
	Number of sections with emboli	0 of 6	1 of 5	1 of 5
Animal 4	Treatment group	Dual API	Dual API	POBA
	Region of sections with emboli	No findings	AL, AP	No findings
	Number of sections with emboli	0 of 6	2 of 5	0 of 5

AA = apical anterior, AL = apical lateral, AP = apical posterior, AS = apical septum, MA = mid anterior, ML = mid lateral, MS = mid septum, MP = mid posterior, LAD = left anterior descending artery, LCX = left circumflex artery, RCA = right coronary artery, RV = right ventricle.

Supplemental Figure

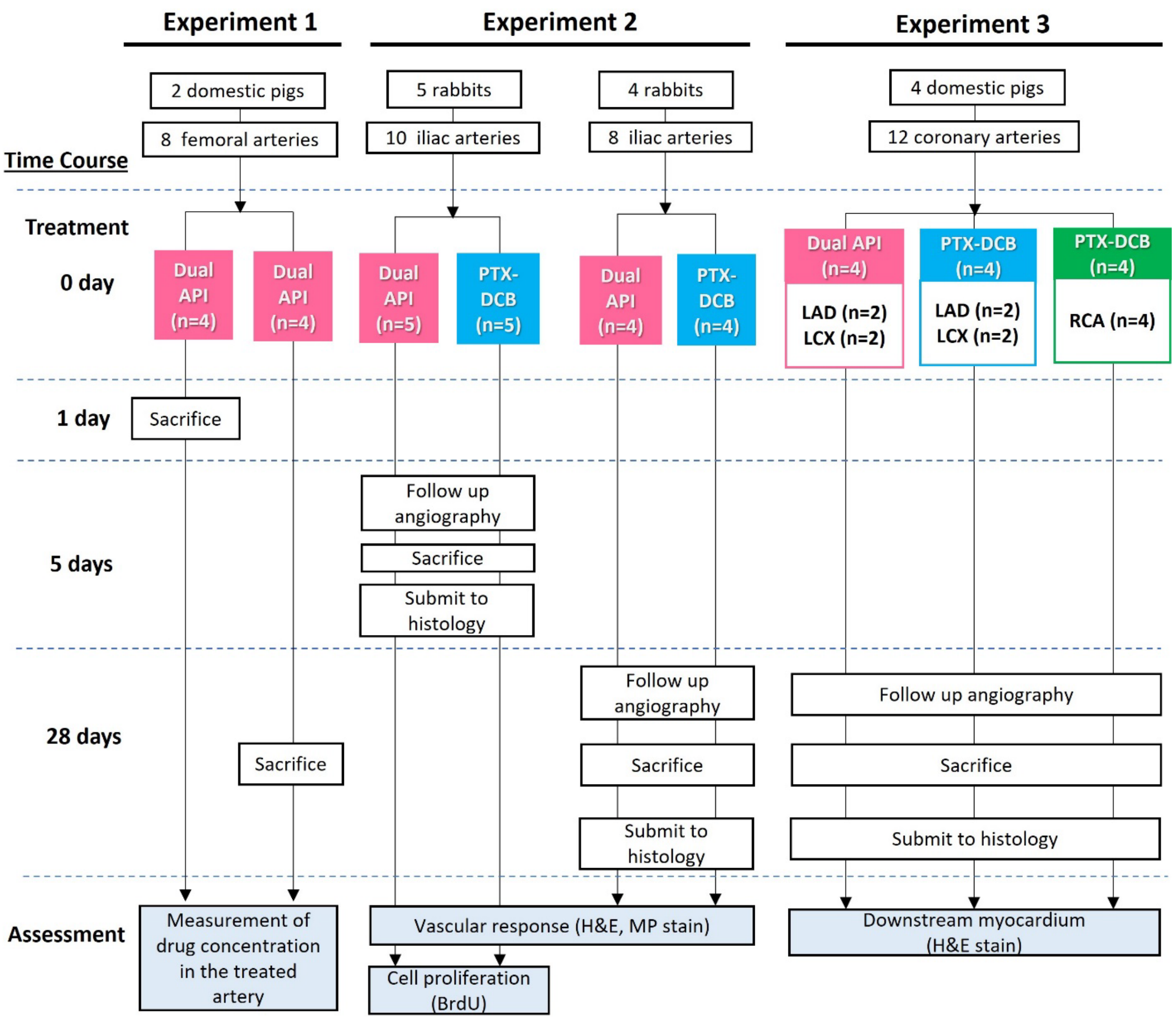

Supplemental Figure 1. Study flow of animal experiments 1 to 3. This study was composed of 3 different animal experiments, including the pharmacokinetic assessment of Dual-API DCB, and the assessment of the vascular response and the effect on downstream myocardium after the treatment with Dual-API DCB or PTX-DCB. Experiment 1 studied pharmacokinetics drug by measurement of drug concentration in the artery 1 day and 28 days after the treatment with Dual-API DCB. Experiment 2 studied vascular response of iliac arteries to DCB treatment using rabbit iliac artery model. The follow-up period was set at 5 days and 28 days after the treatment. The Histology sections were assessed at both timepoints for cell proliferation using BrdU staining at 5 days. Experiment 3 studied downstream myocardium using swine coronary artery model. The follow-up period was 28 days after the treatment.
